# Abrasivity database of different genetic rocks based on CERCHAR Abrasivity Test

**DOI:** 10.1038/s41597-024-03470-2

**Published:** 2024-06-14

**Authors:** Kuidong Gao, Xinyu Wang, Hongxin Wei, Shuxue Wang, Weipeng Xu, Xu Li, Liqing Sun, Hongxiang Jiang

**Affiliations:** 1 College of Mechanical and Electronic Engineering, Shandong University of Science and Technology, Qingdao, 266590 China; 2 China Coal Technology & Engineering Group Shanghai Co., Ltd., Shanghai, 201400 China; 3 College of Mechatronic Engineering, China University of Mining and Technology, Xuzhou, 221116 China; 4 Jiangsu Province and Education Ministry Co-sponsored Collaborative Innovation Center of Intelligent Mining Equipment, Xuzhou, 221008 China

**Keywords:** Sedimentology, Petrology, Mineralogy

## Abstract

Rock abrasivity is one of the main factors affecting the wear of rock-cutting tools, which is usually quantified by the CERCHAR Abrasivity Index (CAI). Researchers and engineers study tool wear and predict tool life based on the CAI of rocks. However, there is still a lack of a dataset on rock properties, especially the abrasivity of various rocks. This paper reports the abrasive dataset of 10 kinds of rocks, including sedimentary rocks, metamorphic rocks, and igneous rocks, with the aid of the CERCHAR Abrasivity Test and digital measurement techniques. The dataset comprises rock abrasivity data, point cloud data for visualization, scratch photos, CERCHAR Abrasivity Test force data, and mechanical properties (uniaxial compressive strength) of rock samples. This dataset facilitates future research on rock abrasivity and rock-cutting tool wear.

## Background & Summary

Mechanically, rock cutting refers to the process of applying force to the rock through the tool to break the rock. Featured by high excavation efficiency and safety, rock cutting is widely used in mining, tunnelling, and construction industries^1,2^. Nevertheless, tool wear is a significant issue in mechanical rock breaking, as it leads to the removal or displacement of cutter material during rock excavation^3^. This wear on the tools greatly increases construction costs, and affects the entire construction period^4^.

Taking tunnel construction as an example, more than 60% of the world’s tunnels are excavated by tunnel boring machines (TBM) (2013)^5^. Studies have shown that when a TBM was adopted to tunnel hard rock strata with huge thrust, the tool would wear rapidly^6^. If construction workers do not replace the worn tools on time, the wear rate of the remaining unworn tools and the number of abnormally worn tools will sharply increase, severely lowering excavation efficiency and tool utilization rate^7^. Even if construction personnel can accurately identify the current wear of cutting tools and replace them on time, tool replacement is still a time-consuming activity that has a significant impact on the daily advance rate (AR) and thus on the time and cost of tunnel construction^8^. According to statistical data, 20% of the project’s construction costs go toward replacing cutters, and their replacement takes up 30% of the project’s total excavation time^9^.

Abrasivity describes the ability of rocks (minerals) to wear (frictionally) the surface of solid materials, which reflects the degree of wear of the material interacting with it^10^.

For any rock excavation project, estimating wear costs is a very challenging task^11^. Research on the abrasivity of rocks is conducive to comprehending the mechanism of rock abrasivity, choosing the applicable excavation equipment^12^, and reducing excavation costs.

**Fig. 1:**
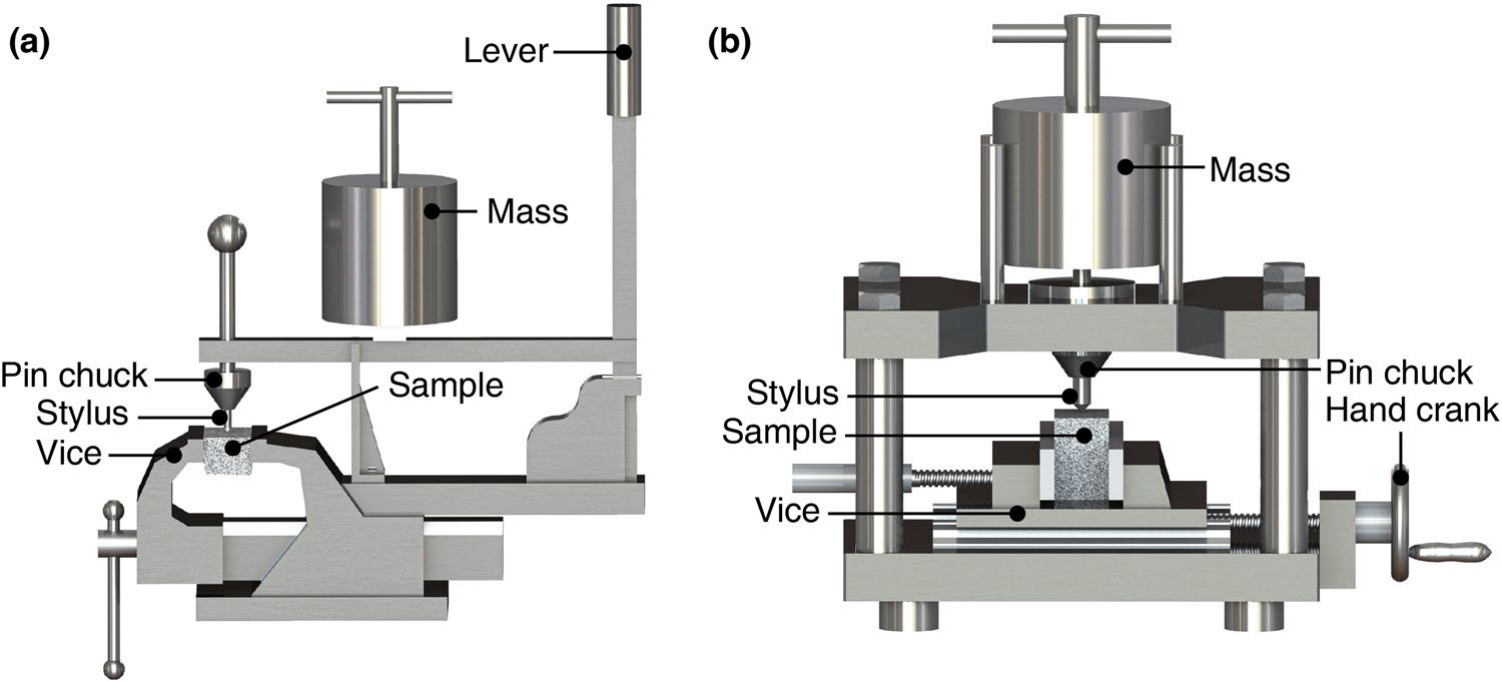
CAI test apparatus. (**a**) Schematic diagram of the CERCHAR-type apparatus and (**b**) Schematic diagram of the modified CERCHAR apparatus employed herein.

CAI is an index for measuring the rock’s abrasivity via the CERCHAR Abrasivity Test^3^. Rock abrasivity matters a lot in the fields of mining, drilling, tunnelling, and construction materials^3,13–15^. In the 1970s, the CERCHAR Abrasivity Test was first developed by the Laboratoire du Centre d’Études et Recherches des Charbonnages (CERCHAR) de France^3^, whose experimental set-up is shown in Fig. 1. Specifically, the CERCHAR-type testing apparatus^3^ is presented in Fig. 1a, and the modified apparatus configuration used here is shown in Fig. 1b.

Although the abrasivity of rocks has been vigorously explored^14–18^, rock samples used for testing come from various mining areas in the world, with different properties. Studies have shown that the abrasivity of rocks is related to the mechanical properties of rocks, and the content of quartz and other abrasive minerals^11,19,20^. Additionally, when the CERCHAR Abrasivity Test is carried out, the normal force applied to the stylus and the skills of the person performing the test will also affect the CAI value^18,21,22^. Therefore, it is necessary to take the aforementioned factors into account rather than just resorting to the CERCHAR Abrasivity Test alone.

**Fig. 2:**
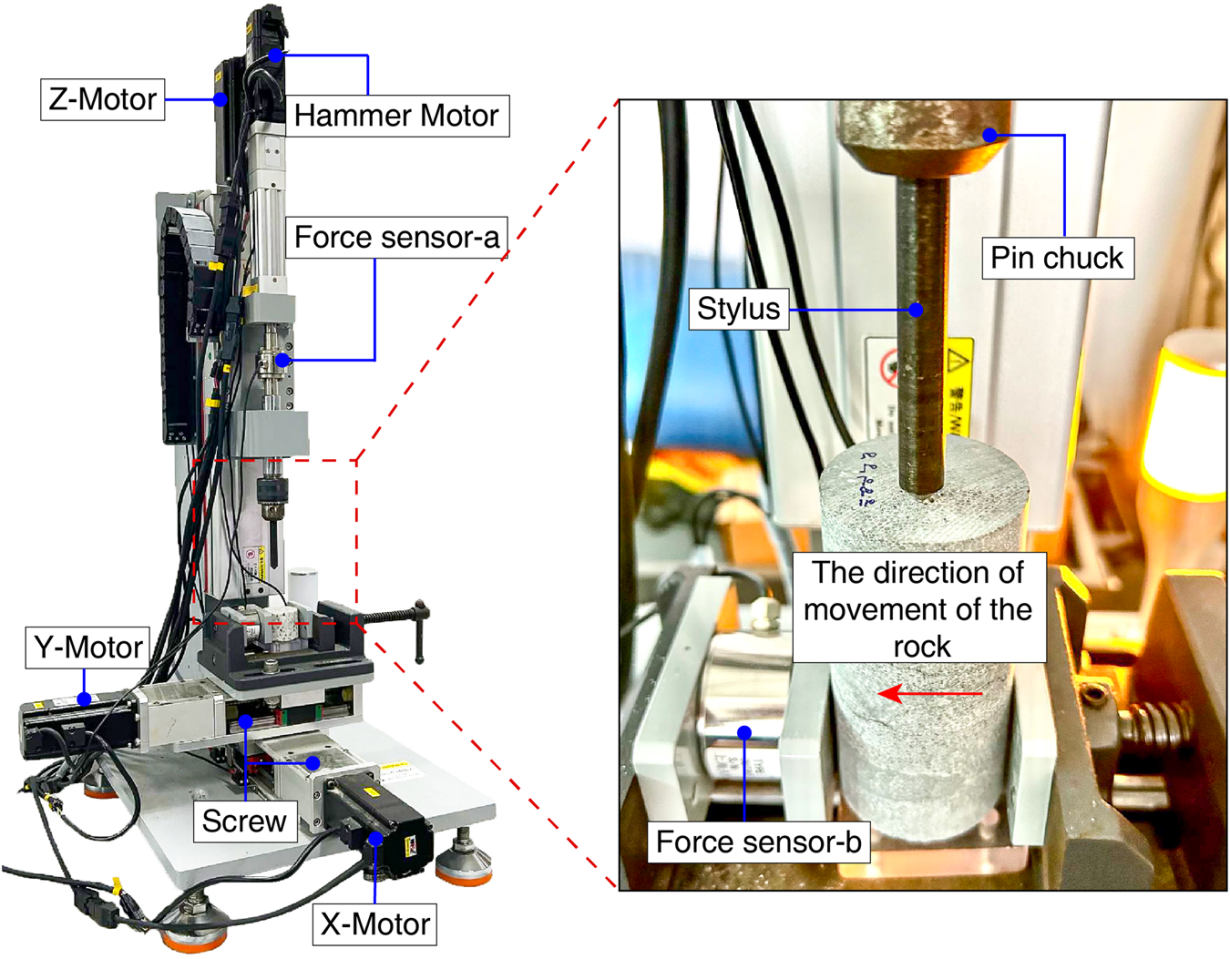
Computerized CAI tester and partial diagram (The principle of the computerized CAI tester is the same as the modified CERCHAR apparatus).

**Fig. 3:**
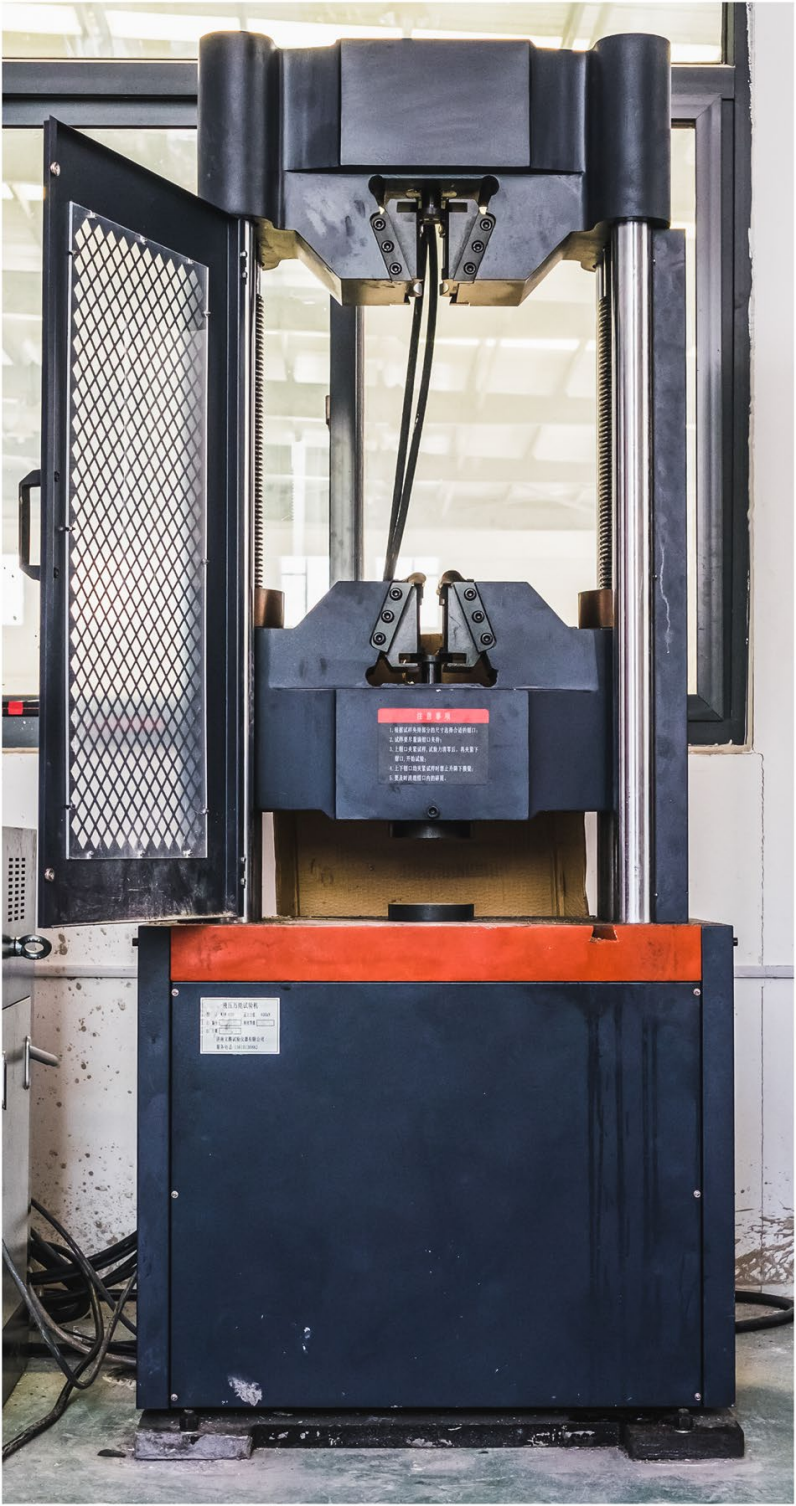
Servo press.

**Fig. 4:**
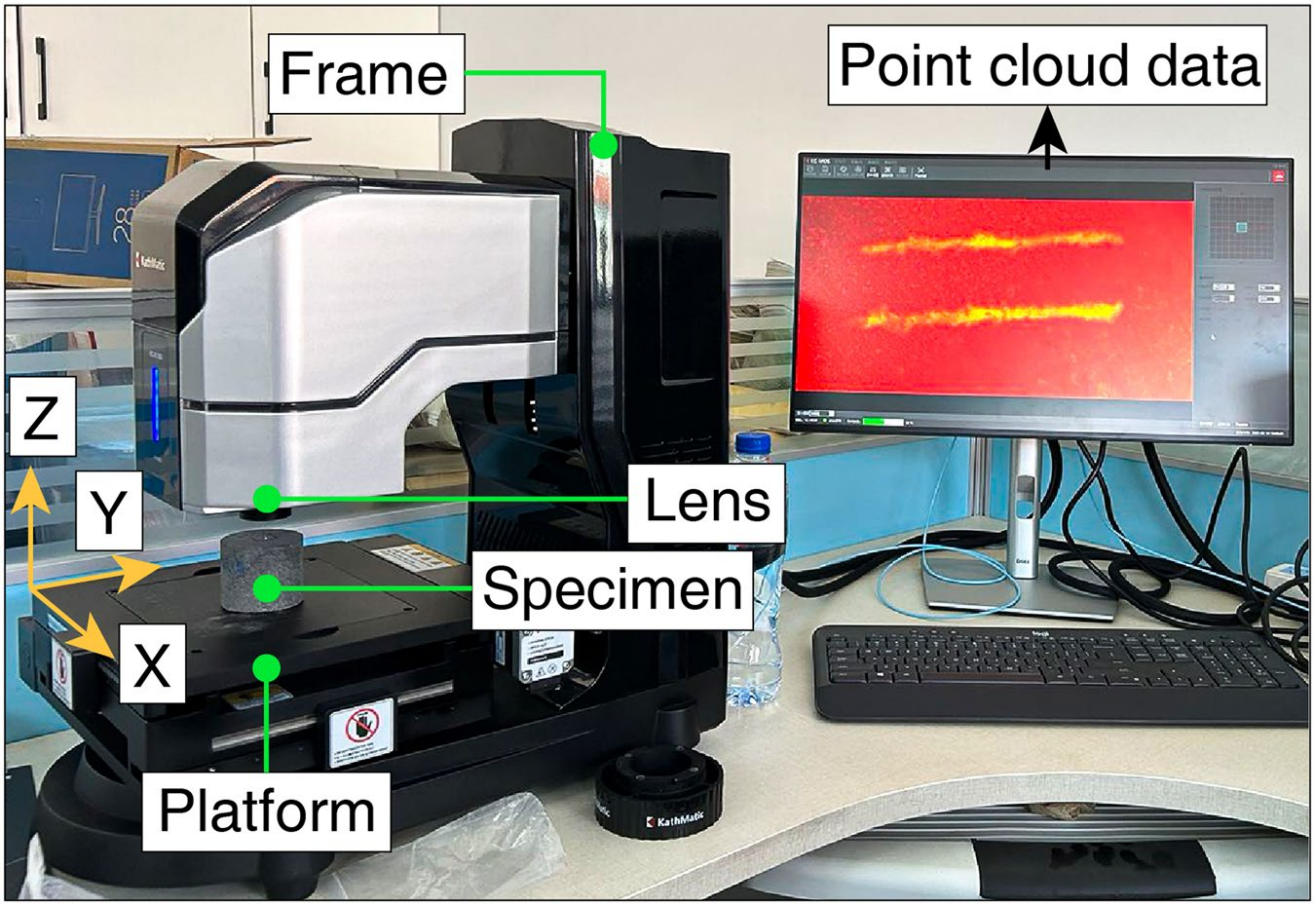
Laser measurement system (laser confocal microscope and computer).

**Fig. 5:**
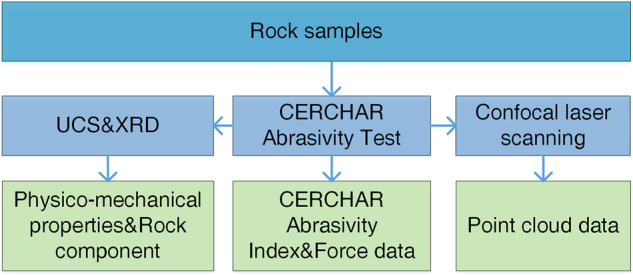
A brief overview of this study.

Taking that into consideration, under four different normal forces, the CERCHAR Abrasivity Test is performed in the present study using the computerized CAI tester (Fig. 2). A servo press (Fig. 3) is utilized for testing the mechanical properties of rock samples. By means of the laser measuring system shown in Fig. 4, the rock’s scratches are measured and recorded in digital camera pictures. The elemental analysis is carried out by X-ray fluorescence (XRF). A brief overview is provided in Fig. 5.

In this study, four different normal forces are applied in the CERCHAR Abrasivity Test to 10 different rocks, including igneous rocks, sedimentary rocks, and metamorphic rocks. Additionally included are the force data collected during the testing, wear data from 40 sets of styluses, pictures, mechanical properties data from rocks, XRF data, and x-ray diffraction (XRD) data. This paper is highly valuable for researching the tool wear, the mechanism of rock abrasivity, as well as the CERCHAR Abrasivity Test.

## Methods

This section describes the rock sample information (source, mechanical properties, composition) used in the CAI test, the specific details of the CERCHAR Abrasivity Test (experimental equipment, test steps, etc.), and the acquisition process of the rock surface point cloud data after the test.

### Test samples

**Fig. 6:**
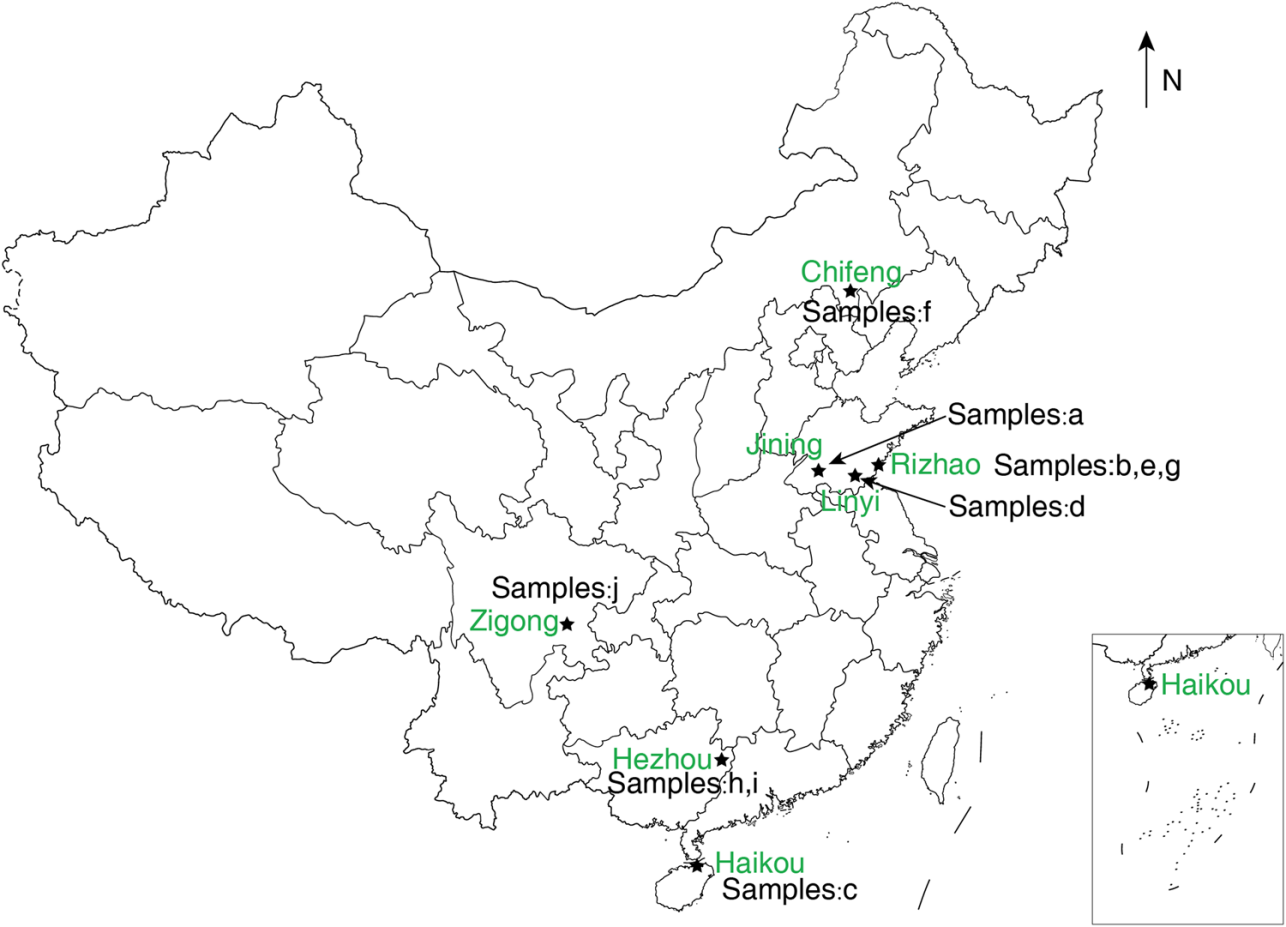
Schematic map of the location of the sampling sites.

In the fields of construction, road, and mining, it is often necessary to break marble, granite, sandstone, and other rocks. In the tunnelling process of some hard rock tunnels, difficult-to-break basalt may also exist. Cutting these rocks will cause wear and tear to the cutting tools. For this rock abrasivity study, we obtained a variety of igneous, sedimentary, and metamorphic rocks from quarries in different parts of China as given in Fig. 6. The rock samples were obtained from open-pit mines within 100 m of the surface. Geological information on each of the mining areas can be found in the literature^23–36^.

The length to diameter ratio of the samples utilized for the test is 2:1 (diameter: 50 mm, length: 100 mm). In the study, all test samples have a homogeneous and isotropic rock matrix that lacks visible pores or fractures. To ensure the comparability of the results, the coring direction of the sandstone is parallel to the normal of the sedimentary surface. Ten rock samples have been selected for the test. Basalt samples are fine-grained rocks (<0.5 mm. Sandstone-green (<0.5 mm), Sandstone-white (<0.5 mm), and Sandstone-purple (<0.5 mm) are all medium sand grains. The Granite-a (0.5–3 mm), Marble (0.5–2 mm), and Granite-c (0.5–3 mm) are medium-grained. The Granite-b (0.5–8 mm), Granite-d (0.5–8 mm), and Marble YPX (0.5–5 mm) are coarse-grained rocks. The naming method and its geological context are shown in Table 1.

**Table 1:** Naming method of rock samples and its geological context.

Serial number	Name of rock	Rock type	Geologic context
a	Granite-a	Igneous rock	Literature^29^
b	Granite-b	Igneous rock	Literature^31–34^
c	Granite-c	Igneous rock	Literature^25^
d	Granite-d	Igneous rock	Literature^30^
e	Sandstone-purple	Sedimentary rock	Literature^31–34^
f	Basalt	Igneous rock	Literature^23,24^
g	Sandstone-green	Sedimentary rock	Literature^31–34^
h	Marble	Metamorphic rock	Literature^26–28^
i	Marble YPX	Metamorphic rock	Literature^26–28^
j	Sandstone-white	Sedimentary rock	Literature^35,36^

#### XRF and XRD detection of rock samples

The composition of the rock samples is detected by X-ray diffractometer and X-ray fluorescence spectrometer, and the XRD data of the rock samples are identified by the software MDI Jade 6.

#### Mechanical test of rock samples

The uniaxial compressive strength (UCS) test, one of the most widely used characterization experiments in rock engineering and engineering geology, is essential for guiding rock breaking practices^37,38^. At ambient temperature, a servo press (600 kN) shown in Fig. 3 is adopted to conduct an uniaxial compressive strength test of rock samples.

**Fig. 7:**
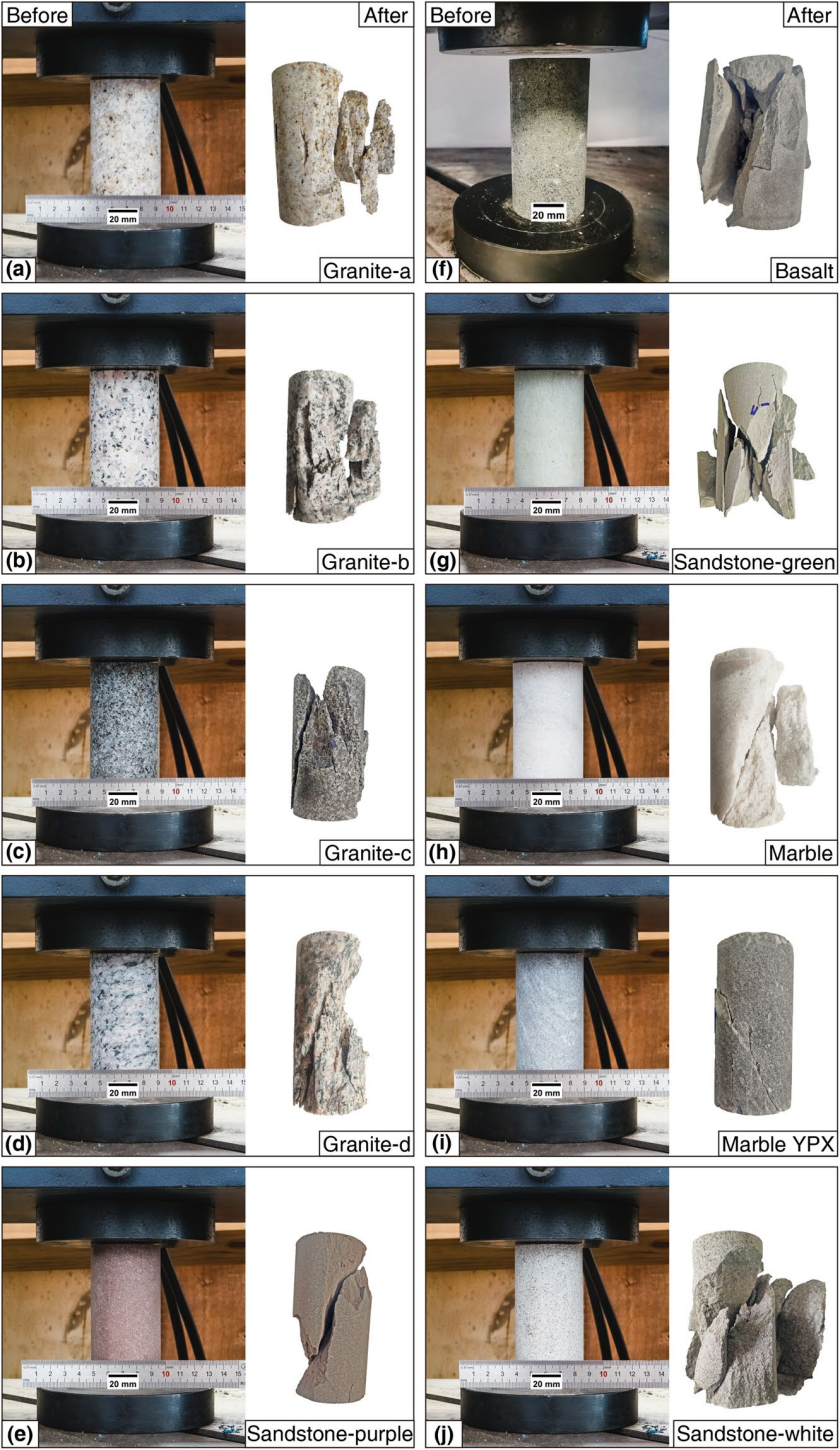
The pictures of rock samples before and after the UCS test. (**a**) The images of Granite-a before and after UCS test, (**b**) The images of Granite-b before and after UCS test, (**c**) The images of Granite-c before and after UCS test, (**d**) The images of Granite-d before and after UCS test, (**e**) The images of Sandstone-purple before and after UCS test, (**f**) The images of Basalt before and after UCS test, (**g**) The images of Sandstone-green before and after UCS test, (**h**) The images of Marble before and after UCS test, (**i**) The images of Marble YPX before and after UCS test, (**j**) The images of Sandstone-white before and after UCS test.

For the loading rate of rocks, the American Society for Testing and Materials (ASTM) recommends employing a stress rate within 0.5–1.0 MPa/s, or loading at a strain rate as constant as possible throughout the entire testing process. The test time recommended by ASTM should be maintained within 2–15 minutes^39^. Based on previous experimental experience, when loading at a rate of 1 kN/s (0.51 MPa/s), the test time is within the recommended time range of ASTM. The rock sample with a length to diameter ratio of 2:1 (diameter: 50 mm; length: 100 mm) is loaded at an axial loading rate of 1 kN/s until the specific rock type fails. Figure 7 shows the pictures of rock samples before and after the UCS test.

The compressive strength *σ*_*u*_ of the rock type is calculated by following equation:

1 $${\sigma }_{{\rm{u}}}=\frac{{\rm{P}}}{{\rm{A}}}$$

*σ*_*u*_ = uniaxial compressive strength (MPa),

*P* = failure load (kN),

*A* = cross-sectional area (mm^2^),

### CERCHAR Abrasivity Test

#### Experimental setup

In this study, the computerized CAI tester is used for testing, as shown in Fig. 3. The principle of the tester is the same as that of the modified CERCHAR apparatus (Fig. 1b). To improve the test accuracy, the displacement of the rock is provided by the X-Motor and Y-Motor, and the normal force is generated by the Hammer Motor. A horizontal force sensor is installed on the vice, and a force sensor is installed above the stylus to monitor the fluctuation of normal force. The tester can achieve a 0–200 N normal force setting, and provide a maximum horizontal force of 500 N. When the force sensor on the vice detects that the force value is greater than 450 N, the tester will enter the protection mode.

#### Stylus

According to the International Society for Rock Mechanics (ISRM)^3^, the diameter of the stylus should be at least 6 mm, and the length between the pin chuck and the rock surface should be at least 15 mm. The stylus used in this experiment has a diameter of 10 mm and a conical angle of 90°. Made of 42CrMo, it is heat-treated to Rockwell hardness (the most widely used hardness index in the metal processing industry) HRC58 ± 1. In each scratch test, one stylus is used only for once.

#### Normal force

The normal force recommended by ISRM is 70 N. However, during this research, it is found that the normal force of the stylus acting on the rock surface affects the obtained CERCHAR Abrasivity Index. To point out this difference for subsequent research, four different normal forces are set, namely, 50 N, 70 N, 90 N, and 110 N.

#### Rock samples

Being cylindrical, the surface of these rock samples is prepared by a water-cooled diamond saw blade. ISRM^3^ does not stipulate the smoothness of the surface of the rock. It can be a rough surface of freshly fractured rock or a sawn-cut surface after processing, which differs only when the CAI value is calculated. Since the sawn-cut surface of the rock is smoother than the rough surface of the freshly fractured rock, the wear-flat diameter of the stylus tested on the sawn-cut surface is smaller than that of the rough surface, and the calculated CAI value needs to be corrected^3^ by Eq. (4).

#### Testing procedure

**Fig. 8:**
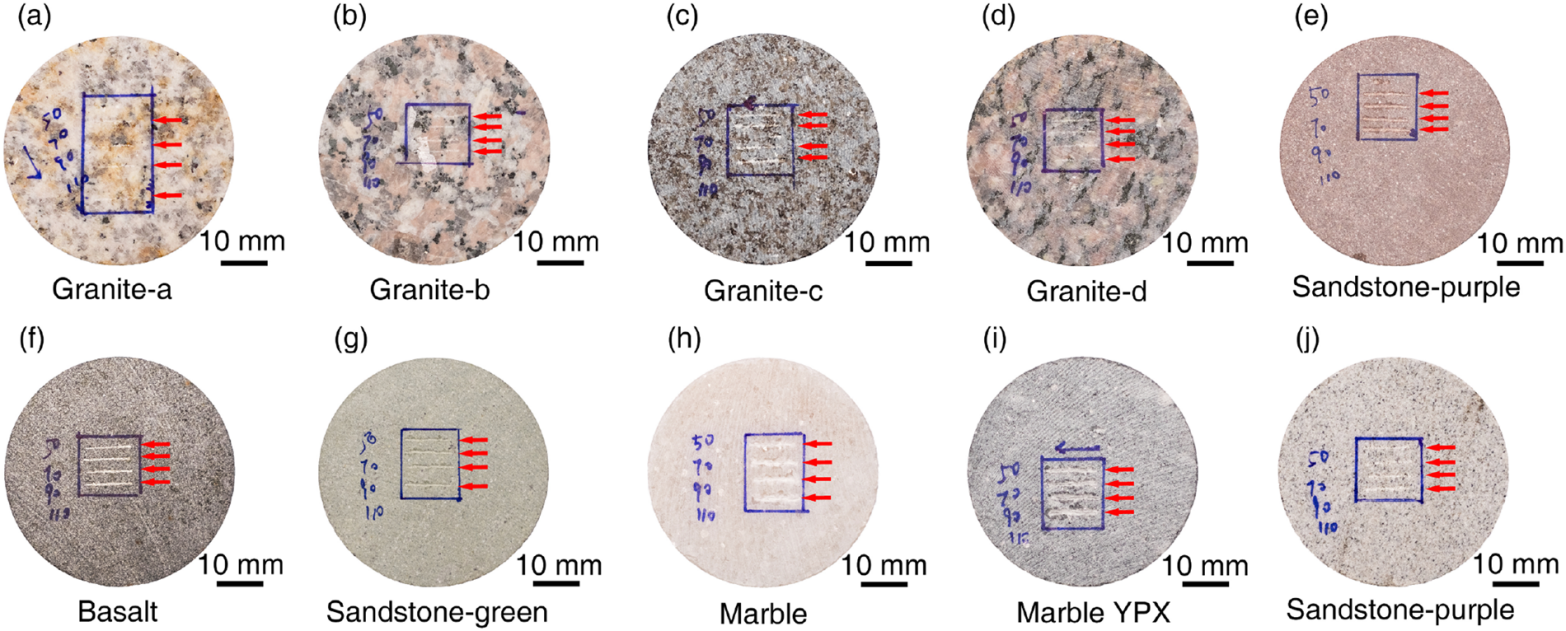
Schematic diagram of the location of rock scratches.

A total of 40 CERCHAR Abrasivity Tests of rocks are performed, with 4 tests for each rock. Different normal forces are applied to the same rock sample. As shown in Fig. 8, from top to bottom are scratches under the normal forces of 50 N, 70 N, 90 N, and 110 N, respectively.

Under the microscope, it is checked whether there are obvious damage marks at the tip of the stylus, and the damaged stylus is not used in this study. Next, the rock is clamped to the vice, and the pre-tightening force is applied to the sample to prevent it from shaking freely. By adjusting the control handle to make the stylus contact with the rock surface, the normal force between the stylus and the rock surface is slightly adjusted so that it is greater than 20 N and less than the target normal force to ensure that the tip of the tool bites into the rock. For coarse-grained rocks, to reduce the serious impact of the rebound of the stylus on the rock surface during the calculation of the CAI value in the test, the rock-moving speed should be reduced to seconds per mm^11^. To ensure uniformity of test results, the moving speed on the control panel of the tester is set to be 10 mm/min. After starting the test switch, the tester automatically adjusts the force between the grinding needle and the rock to the target normal force through the control system and drives the rock to move horizontally for a distance of 10 mm. At this time, the test stops. Then, the Z-Motor is started, and the stylus is lifted from the rock surface to remove the stylus from the pin chuck carefully.

#### Stylus measurement and CAI calculation

**Fig. 9:**
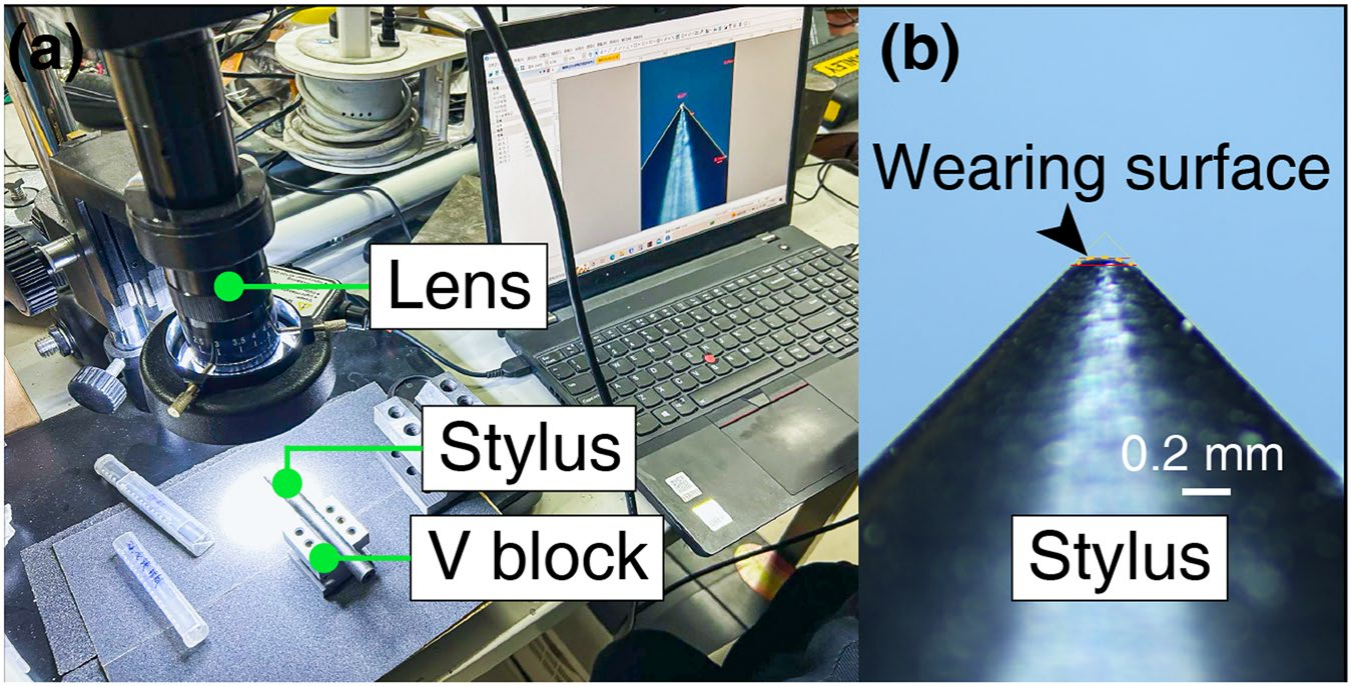
Digital Microscope.

**Fig. 10:**
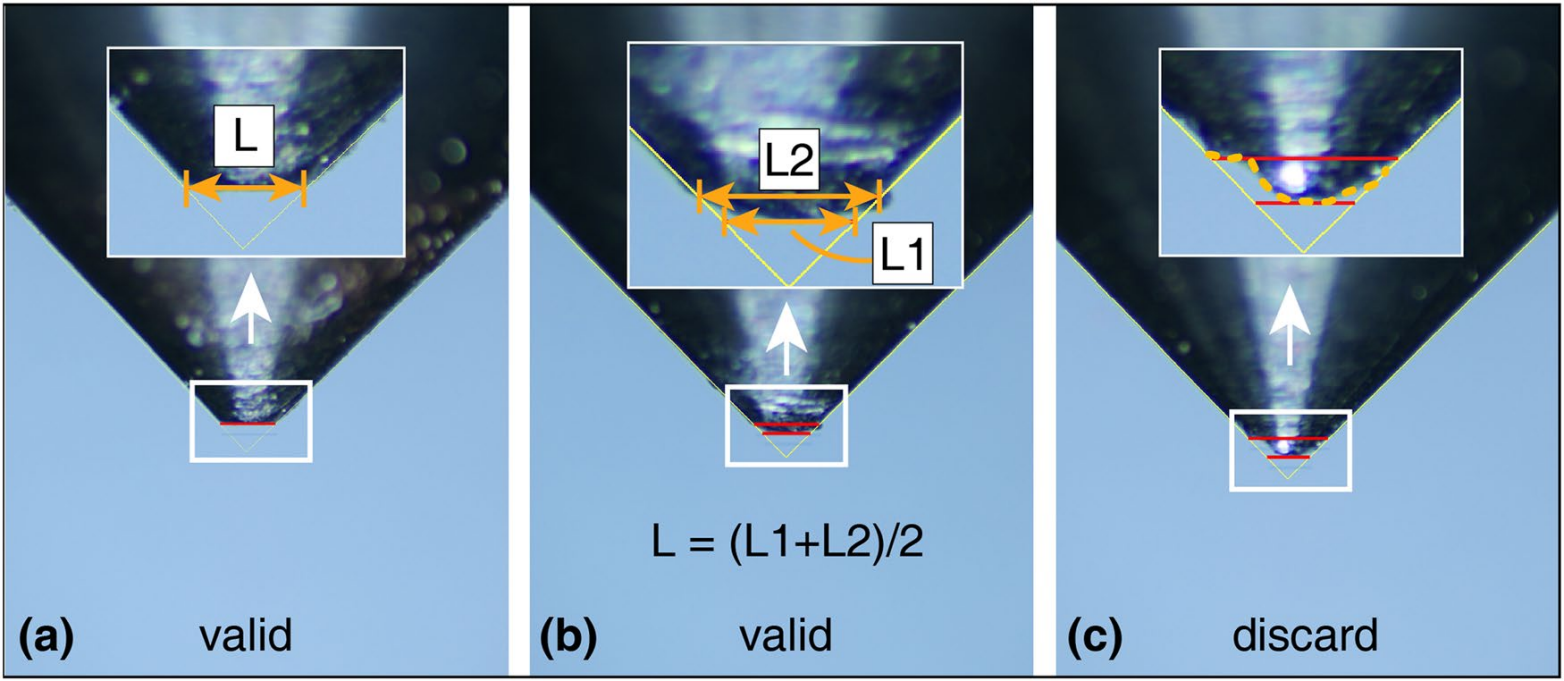
Stylus Measurement Standards (taking the stylus in this study as a case). (**a**) The wear surface of the stylus is flat and available, (**b**) The stylus has an arc surface, in this case, two values L_1_, L_2_ should be measured and then the average value should be taken, (**c**) When asymmetric wear occurs, retesting should be performed; otherwise, it will bring about greater measurement error.

The rock fragments on the tip of the stylus are cleaned up by compressed air and placed on a V block. A professional scale is utilized to calibrate the digital microscope (Fig. 9) and measure the stylus. According to the ISRM^3^ recommendation, the wear flat diameter of the stylus should be measured from the top. However, the burrs at the tip of the stylus may extend beyond the wear flat, which in turn affects the measurement values. To ensure the accuracy of the measurement results, the side view measurement is used, and the measurement standard from ISRM is shown in Fig. 10.

Based on the measurement results of the wear flat diameter, the CAI value is calculated by Eq. (2)

2 $${\rm{CAI}}={\rm{L}}\times 10$$

The stylus hardness obtained from different batches of steel may not be consistent during the heat treatment process. According to the ISRM^3^ recommendation, when a stylus test other than Rockwell hardness HRC55 ± 1 is adopted, the CAI value should be corrected to the one obtained under Rockwell hardness HRC55 ± 1, as converted by Eq. (3):

3 $${\rm{CAI}}{\prime} =\frac{0.415{{\rm{CAI}}}_{\left(x\right)}}{\left(1-0.0107x\right)}$$

wherein CAI(*x*) is measured as the CAI value using a stylus with a hardness of HRC*x*.

According to ISRM^3^, the rock surface is a sawn-cut surface and the measurement values should be corrected by Eq. (4)**:**

4 $$L=1.14{L}_{s}$$

wherein *d*_*s*_ is the wear flat diameter of the stylus measured after the test on the sawn-cut surface.

### Acquisition of point cloud data of rock surface scratches

The point cloud data are collected by using a laser measurement system, as depicted in Fig. 4. With a model of KC-X1000, the microscope has a sampling frequency of 200–4,500 Hz. Configured with the lens KC-H030, the microscope has a range of ±600 μm with a resolution of 10 nm.

**Fig. 11:**
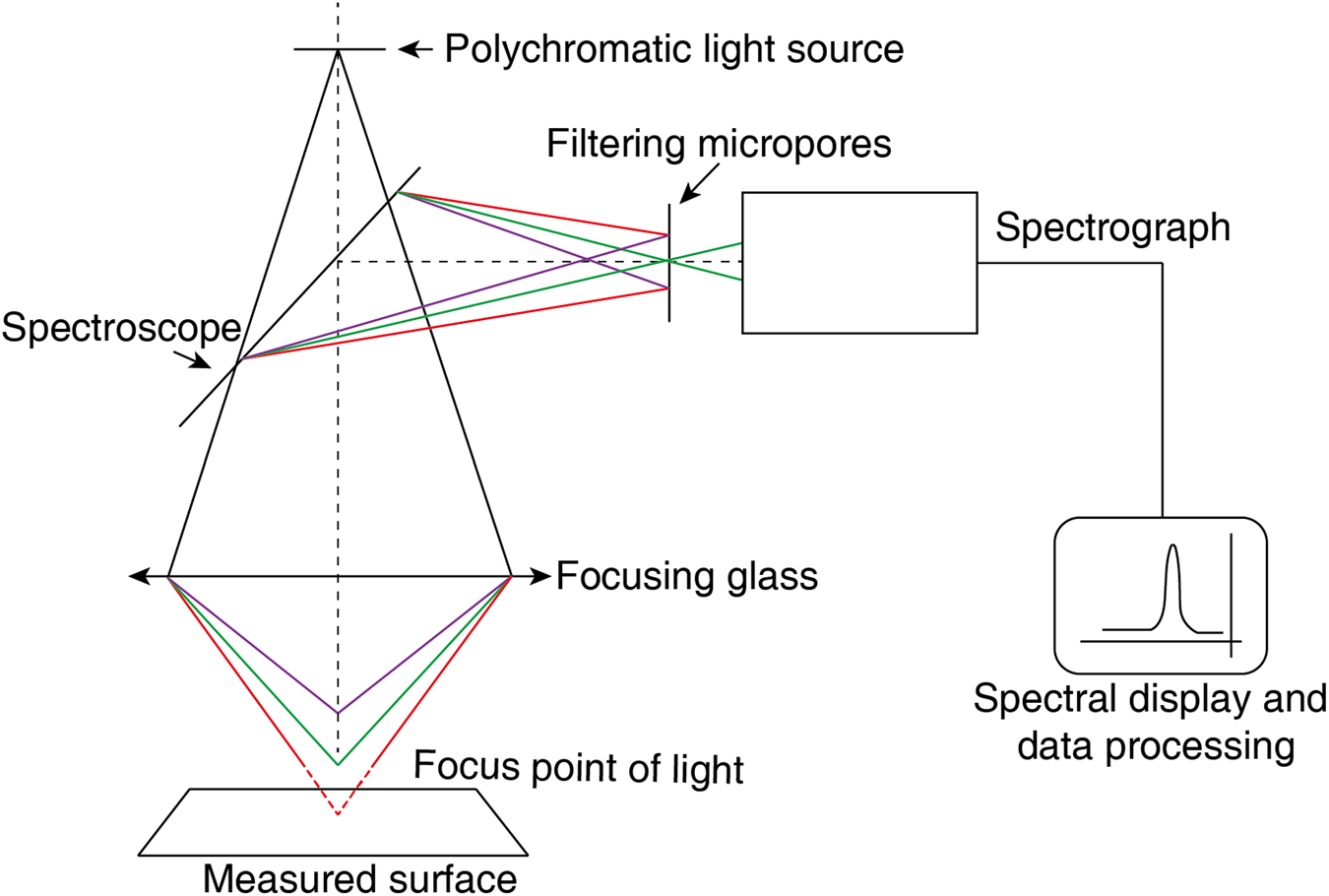
Schematic diagram of laser confocal microscope.

Figure 11 illustrates the schematic diagram of the laser confocal microscope. A special type of luminescent material, which is excited by a blue laser with a wavelength of 450 nm, is used as the light source. This material emits high-intensity polychromatic light, which greatly enhances the spectral confocal technology’s Z-axis resolution, up to 2 nm. The wavelength resolution of the spectral detector is as high as 0.015 nm, and the signal-to-noise ratio is greater than 15,000: 1, endowing it with strong anti-interference capabilities.

After the CERCHAR Abrasivity Test, compressed air is used for carefully cleaning up the rock powder under the scratching of the rock surface, and then the powder is placed it on the XY mobile platform of the laser confocal microscope for detection. The positioning accuracy of the XY mobile platform is ± 1 μm, and the repeated positioning accuracy is 0.5 μm; the resolution is 100 nm, and the displacement stroke is 100 × 100. The acquisition of point cloud data is automatically completed by the laser measurement system (Fig. 4). After the detection is completed, the rock is taken down, and then the point cloud data are exported from the device.

The volume of rock debris can be calculated with the use of point cloud data, and the wear ratio (rock cutting volume/tool wear volume) of different types of rocks can be obtained by combining the wear volume of the stylus (calculated by the wear size and cone angle of the stylus), providing a basis for the prediction of rock-breaking tool life. Under different normal force conditions, the rock surface shows different scratch morphologies. Through the visualization of point cloud data, the differences can be intuitively identified. Combined with CAI value, rock composition, mechanical properties (UCS), etc., the wear effect of a certain type of rock on cutting tools can be analyzed. In addition, during CAI experiments, researchers can also extract coordinate data on a certain line for post-processing based on point cloud data. For example, in the vertical or parallel scratch direction, the depth data under different normal forces are extracted.

## Data Records

The dataset is available at Figshare^40^ (10.6084/m9.figshare.23939310).

There are 6 folders in the dataset (containing data on the composition, mechanical properties, point cloud data of rock scratches, and abrasivity data of 10 types of rocks), which are uploaded with the paper.

The file “Force Data” shows the force data collected during the CERCHAR Abrasivity Test, including the original file, the processed file, and Readme file.

The file “Laser Point Cloud Data” is a point cloud file scanned by a digital laser confocal microscope, which contains the original file and the file processed using Cloudcompare _ v2.13.

The file “Rock composition” contains the XRD and XRF files of the rock.

The file “Rock UCS” contains rock images before and after rock mechanics testing.

The file “Scratch Pictures” contains scaled rock scratch images from the CERCHAR Abrasivity Test.

The file “Stylus Measurement” contains images of stylus’ wear flat diameters measured using digital microscope.

## Technical Validation

### Experimental error analysis

The rock samples used in this study are taken from large rock masses and subjected to non-uniformity checks before the test. In the CAI scratch test, abrasion means that in essence the material of the stylus is forced to be removed or displaced during the cutting of rocks by the stylus. For rocks with high strength and coarse particles, the collapse of rock debris is inevitable, which may create pits on the rock surface. In the process of obtaining point cloud data, there may be a very small number of points whose spatial positions exceed the depth of field of the laser confocal microscope. Due to the automatic acquisition and recording of experimental data through the tester, the data error obtained may come from the equipment itself. To obtain more accurate measurement values, high-precision testing instruments and analysis methods are recommended to analyze and process data, such as filtering point cloud data to reduce experimental errors.

### Reliability of testing results

Each test is completed using standardized measuring instruments, and the data are of high accuracy and reliability. The signal is generated by the sensor on the tester and collected by the data acquisition system. The instruments for collecting data are high-precision instruments with mature technology. Before each test, the equipment with signal recording function is calibrated, and the surrounding electric field, magnetic field and severe vibration are also isolated.

## Usage Notes

When processing point cloud data, the reader may choose any other software that can process point cloud data. If a smoother 3D model is obtained, filtering algorithms can be appropriately used for processing.

## Data Availability

The equipment used for laser scanning models is from KathMatic company. The software used for point cloud data visualization is Cloudcompare_ V2.13, which is a very convenient software for processing point cloud data, can run on Windows, MacOS, and Linux systems, and is also open source. The software used for force data processing is Microsoft Excel 2016 and Origin 2018. The microscope used to photograph stylus is the AO-3M180 digital microscope. The press used for mechanical property testing is a 600 kN electro-hydraulic servo testing machine. The image is captured using a digital camera and macro lens.
